# Cholestatic hepatitis in a patient with typhoid fever - a case report

**DOI:** 10.1186/1476-0711-10-35

**Published:** 2011-10-08

**Authors:** Eranda C Ratnayake, Chrishan Shivanthan, Bandula C Wijesiriwardena

**Affiliations:** 1 Department of Medicine (Ward 45), the National Hospital of Sri Lanka, (Regent Street), Colombo, Sri Lanka

## Abstract

Typhoid fever is a very common infectious disease, particularly in developing countries such as Sri Lanka. Although multiple organs are known to be affected by the disease, hepatic involvement could be considered the most important as studies have showed that it is associated with a higher relapse rate. We report a young patient who presented with fever and jaundice and found to have cholestatic hepatitis secondary to typhoid fever.

## Introduction

Typhoid fever is known to cause a wide range of hepatic complications [1]. However, cholestasis secondary to typhoid fever has only been reported in a few instances [2,3]. The pathophysiology of liver dysfunction due to typhoid infection is not yet fully understood. Demonstration of intact *Salmonella typhi *in liver tissue of patients with typhoid fever suggests that there is an interplay of the micro organism factors and the immunity which cause liver injury [3,4].

## Case report

A 33 year old male sought hospital care after anorexia, and fever for 10 days followed by a vague right hypochondrial discomfort, vomiting and jaundice for 3 days with dark urine and some reduction in urine output. There was an associated diarrohoea with yellow stools. He did not complain of pruritus. He did not give a contact history with muddy water and denied blood transfusions, promiscuity or intravenous drug abuse.

He had been employed as a driver and had no significant past medical history or travel history to a malarial endemic area. He consumed alcoholic beverages only at social functions and denied any recent binges. He was not using any medication prior to this admission including antibiotics.

Examination revealed an ill looking patient with deep icterus with mild dehydration but without any scratch marks. He had no stigmata of chronic liver disease or evidence of encephalopathy. Fever was documented and no lymphadenopathy was detected. There was tender firm hepatomegaly with moderately enlarged soft spleen. The gall bladder was not palpable. Cardiovascular, respiratory and neurological examination was normal.

The urine full report showed bile in the urine with normal levels of urobilinogen. Full blood count showed a borderline leucopaenia - 4.8 × 10^3^/μl with 51% neutrophils and 44% lymphocytes. The platelet count was 175 × 10^3^/μl and never dropped significantly throughout his hospital stay. The initial ESR was 35 mm/h and the CRP was elevated 96 mg/l. The blood picture showed only mild rouleaux formation.

The liver biochemistry showed normal albumin levels with elevation of AST and ALT which were 120 units/l and 240 units/l respectively The alkaline phosphatase was 1500 units/l, Indirect Bilirubin was 1 mg/dl and the Direct Bilirubin was 10 mg/dl (Table 1). The serum electrolytes and renal function were within normal limits.

**Table 1:** Liver biochemistry time-line

	Day 1	Day 5	Day 10	Day 20	Day 30
AST (U/L)	120	125	110	70	40

ALT (U/L)	240	250	255	100	70

ALP (U/L)	1500	1600	1400	600	250

Direct Bilirubin (mg/dl)	10	16	8	4	2

Indirect Bilirubin (mg/dl)	1	2	2	1	0.6

Albumin (g/l)	37	38	36	36	40

(Normal values - AST < 45, ALT < 50, ALP < 200, Direct bilirubin < 0.6, Albumin 35-50)

The ultrasound scan of the abdomen showed an enlarged liver at 17.5 cm with a coarse echo pattern and a markedly dilated gall bladder with normal wall thickness. The intra and extra-hepatic bile ducts were not dilated nor were any calculi visualized. There was splenomegaly at 15.5 cm.

Blood culture, urine culture, serology studies for viral hepatitis, leptospirosis and dengue ere dispatched and, he was empirically treated with IV crystalline penicillin as leptospirosis was endemic in the region despite the negative contact history.

His fever continued for four days post-hospital admission and the icterus progressively worsened despite treatment for suspected leptospirosis. On serial monitoring the liver biochemistry showed further derangement with marked increase of direct bilirubin to a maximum of 16 mg/dl and alkaline phosphatase to a maximum of 1600 Units/L and mild increase of transaminases, with a maximum AST of 125 Units/L and ALT of 255 units/L (Table 1).

The Leptospira microscopic agglutination test was equivocal and hepatitis serology was negative for Hepatitis A, B and C. The Dengue IgG was positive in low titres however with a negative IgM.

The patient refused to give consent for a percutaneous liver biopsy.

His blood culture yielded a growth of *Salmonella typhi *after 5 days incubation, with antibiotic sensitivity to Ciprofloxacin (MIC < 0.5 mg/L) and Cefotaxime (< 0.25 mg/L). The strain was resistant to Ampicillin (MIC > 1 mg/L) and Chloramphenicol (MIC > 2 mg/L)

He was started on IV Cefotaxime 2 g three times a day for 3 days. Oral Azithromycin 1 g daily for 7 days was also added. This combination of antibiotics is the usual recommendation by the department of microbiology of our hospital.

He demonstrated marked clinical improvement with defervescence in 48 hours and gradual resolution of jaundice and constitutional symptoms over the next 7 days. The liver biochemistry subsequently showed decline of cholestasis with improvement of transaminases (Table 1) by day 30. The persistence of mild enzyme derangement at this stage could be due to residual hepatic damage. Further monitoring of liver enzymes was planned to detect chronic liver disease.

He was discharged after seven days of hospital treatment with oral Cefixime and Azithromycin. He returned one week later and was clinically well with just a tinge of jaundice with absence of hepatomegaly and splenomegaly. The liver biochemistry was gradually improving and by day 30 of the illness was just about baseline.

## Discussion

Typhoid fever or enteric fever is a major health burden in developing countries. It is caused by *Salmonella typhi *and *Salmonella paratyphi*. The faeco-oral route is the commonest mode of transmission and poor sanitation and reduced access to clean drinking water increases its prevalence and incidence.

The absence of any other underlying cause for the cholestasis in our patient, including alcohol, medication, other viral causes of hepatitis as well as the prompt response to treatment suggests that the cholestatic hepatitis in this instance was indeed due to typhoid fever.

Our patient had certain similarities with the previously reported cases of typhoid cholestatic hepatitis, including presentation and resolution of liver derangement with treatment. Although third generation Cephalosporins are not used commonly in treating typhoid fever in most developed countries, its use in developing countries is highlighted in our case as well as in one other case [3].

It can spontaneously resolve after a subclinical course of event or proceed to cause mainly constitutional symptoms and even complications such as bowel perforation which can lead to haemorrhage, peritonitis and even death. Rare complications whose incidences are reduced by prompt antibiotic treatment include disseminated intravascular coagulation, hemophagocytic syndrome, pancreatitis, hepatic and splenic abscesses and granulomas, endocarditis, pericarditis, myocarditis, orchitis, hepatitis, glomerulonephritis, pyelonephritis and hemolytic uremic syndrome, severe pneumonia, arthritis, osteomyelitis, and parotitis [4,5].

Hepatic involvement of typhoid fever was reported by William Osler in 1899 [6]. The pathophysiological mechanism by which *Salmonella *produces hepatic dysfunction, although not fully known as yet is postulated to be either due to direct invasion or by endotoxaemia with immune mediated liver damage [1]. The liver involvement can vary from mild elevation of aminotransferases and alkaline to a levels indistinguishable from acute viral hepatitis [3]. A predominantly cholestatic picture however has been only documented in literature very rarely [2,3]. The exact pathophysiology of cholestasis in preference to hepatitis needs a deeper evaluation and tissue studies of patients with Typhoid fever with hepatitis and cholestasis are necessary.

The treatment of typhoid is governed by antibiotic sensitivity and local resistance patterns [7]. There is an alarming rise of *Salmonella typhi *strains which are considered Multi-Drug Resistant, particularly in the Indian Subcontinent [8]. Stringent adherence to local microbiological protocols would go a long way in preventing the worsening of this catastrophic phenomenon.

## Conclusions

Although rare, cholestatic hepatitis should be recognized as an associated manifestation of typhoid fever, especially in typhoid endemic countries such as those in South Asia.

## Consent

Written informed consent was obtained from the patient for publication of this case report. A copy of the written consent is available for review by the Editor-in-Chief of ***Annals of Clinical Microbiology and Antimicrobials***.

## Competing interests

The authors declare that they have no competing interests.

## Authors' contributions

REC carried out the literature search and drafted the manuscript; WBC did the critical revision for important intellectual content in the manuscript and given the final approval of the version to be published; SC helped substantially in literature search and drafting the manuscript. All authors read and approved the final manuscript
